# Data on organochlorine concentration levels in soil of lowland paddy field, Kelantan, Malaysia

**DOI:** 10.1016/j.dib.2018.08.178

**Published:** 2018-09-03

**Authors:** Bibie Evana Osman, Wan Mohd Afiq Wan Mohd Khalik

**Affiliations:** a School of Marine and Environmental Sciences, Universiti Malaysia Terengganu, 21030 Kuala Nerus, Terengganu, Malaysia; b Centre for Water Research and Analysis, Universiti Kebangsaan Malaysia, 43600 Bangi, Selangor, Malaysia

## Abstract

The main goal of this research work is to measure the concentration levels of organochlorine residue in soil. The potential health risk of this pollutant on human was also determined. 10 samples were taken from a lowland paddy field situated in Kelantan, Malaysia. Physical parameters namely soil pH, organic carbon content, water content and particle size were identified to evaluate the quality of soil from the agriculture site. Soxhlet extraction and florisil clean-up process were applied to isolate 10 targeted organochlorine compounds prior to the final determination using a gas chromatography-electron capture detector. Soil from the lowland has characteristics such as slightly acidic, low organic carbon content, high water content and texture dominated by the sandy type. Concentration levels of six detected organochlorine pesticides were calculated in µg/kg. Hazard quotient value in all samples was less than the acceptable risk level HQ ≤ 1, thus reflecting the status of soil in the subjected area as unlikely to pose any adverse health effects.

**Specifications table**

**Table:** 

Subject area	*Environmental Sciences*
More specific subject area	*Soil pollution*
Type of data	*Tables and figures*
How data were acquired	*Gas chromatography-electron capture detector (organochlorine pesticide analysis), pH (soil pH analysis), water content (Carbolite bench top oven), organic content (laboratory furnace)*
Data format	*Raw and analysed*
Experimental factors	*Samples were extracted by Soxhlet extraction and clean-up using florisil*
Experimental features	*Organochlorine pesticide analysis*
Data source location	*Machang, Kelantan state, Malaysia*
Data accessibility	*Data available within the article*

**Value of the data**

• The data serve new information about soil characteristics from a lowland paddy field.
• The data provide latent information on the occurrence of banned pesticide in Malaysian agriculture soil.
• The data of hazard quotient show that soil in the subjected area poses less risk toward human health.

## 1 Data

**Fig. 1:**
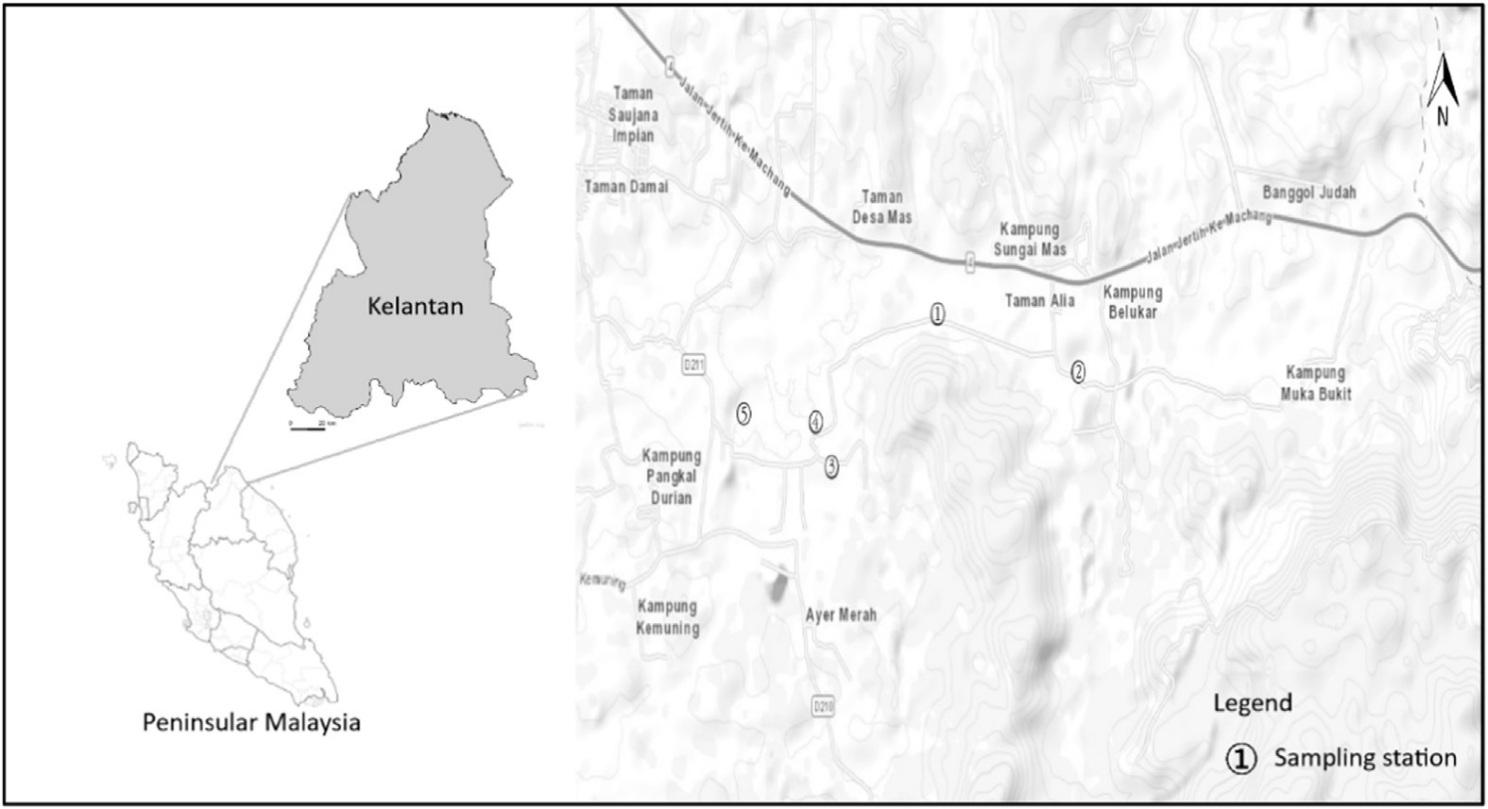
The map of sampling station located in Machang, Malaysia.

Location and coordinates for each sampling point are shown in Fig. 1 and Table 1, respectively. A summary of the soil quality characteristics for the collected soil samples is presented in Table 2. All samples showed similar characteristics, which are slightly acidic, low organic carbon content, high water content and texture dominated by the sandy type. No significant difference was reported between two fields of the survey datasets except water content (*p* < 0.05). The increment of water content was linked to rainy season during 2nd sampling survey. Concentration ranges and mean value of detected organochlorine pesticide are tabulated in Table 3. Only six detected pesticides are presented in the data article. Table 4 explicates that the hazard quotient value obtained is unlikely to pose any adverse health effects through the ingestion route.

**Table 1:** The coordinate of sampling point.

**Station**	**Latitude (N)**	**Longitude (E)**	**Altitude (m)**
S1	5°45′14.1″	102°15′51.7″	52.42
S2	5°45′01.9″	102°16′40.0″	47.79
S3	5°44′41.8″	102°15′15.2″	47.50
S4	5°44′51.2″	102°15′09.6″	42.87
S5	5°44′53.1″	102°14′45.0″	47.93

**Table 2:** Soil characteristics of lowland samples.

**Station**	**pH**		**OC (%)**		**WC (%)**		**Clay (< 2 µm)**	**Silt (< 20 µm)**	**Sand (< 200 µm)**
	**1st**	**2nd**	**1st**	**2nd**	**1st**	**2nd**			
S1	6.50	6.13	2.39	3.33	32.10	69.14	3.01	13.98	82.93
S2	5.56	6.22	3.44	3.32	30.22	53.40	7.07	10.93	81.90
S3	6.17	6.29	2.40	4.91	20.44	66.27	1.55	6.32	82.83
S4	5.97	6.10	3.81	4.57	32.89	56.50	2.34	10.88	86.61
S5	6.30	6.20	2.98	3.52	26.58	65.79	1.65	5.59	92.67

OC: organic carbon, WC: water content.

**Table 3:** Concentration range and mean of detected pesticides.

**Compound**	**Linearity equation**	**1st field survey**		**2nd field survey**	
		**Range (µg/kg)**	**Mean ± SD (µg/kg)**	**Range (µg/kg)**	**Mean ± SD (µg/kg)**
α HCH	*y* = 300*x*−0.95	< LOD–7.34	4.43 ± 1.57	< LOD–4.85	4.43 ± 0.36
β HCH	*y* = 100*x*−0.07	< LOD–3.12	3.05 ± 2.19	< LOD–5.12	3.35 ± 2.50
γ HCH	*y* = 300*x*−0.32	< LOD–3.73	2.13 ± 1.37	< LOD–2.13	2.12 ± 0.01
δ HCH	*y* = 100*x*−0.01	< LOD–1.95	1.95 ± 0.60	< LOD–1.75	1.72 ± 0.03
4-4′-DDT	*y* = 70*x*−0.06	0.49–5.24	1.50 ± 0.21	0.09–1.52	0.20 ± 0.01
Endosulfan sulphate	*y* = 200*x*−0.64	< LOD–0.03	0.02 ± 0.01	< LOD–0.02	0.02 ± 0.01

LOD: Limit of detection, LOD α HCH (0.02), β HCH (0.02), γ HCH (0.02), δ HCH (0.03), endosulfan sulphate (0.01) express in µg/kg.

**Table 4:** Chronic daily intake and hazard quotient of detected pesticides.

**Compound**	**Adult**			**Children**		
	**CDI**	**HQ**	**Health risk**	**CDI**	**HQ**	**Health risk**
α HCH	8.58 × 10^−^^9^	2.86 × 10^−^^5^	No	9.61 × 10^−^^8^	3.20 × 10^−^^4^	No
β HCH	3.31 × 10^−^^9^	1.10 × 10^−^^5^	No	9.70 × 10^−^^8^	3.23 × 10^−^^4^	No
γ HCH	1.16 × 10^−^^9^	3.89 × 10^−^^6^	No	5.62 × 10^−^^8^	1.87 × 10^−^^4^	No
δ HCH	3.34 × 10^−^^9^	1.12 × 10^−^^5^	No	3.75 × 10^−^^8^	1.25 × 10^−^^4^	No
4-4’-DDT	1.34 × 10^−^^9^	2.68 × 10^−^^6^	No	4.59 × 10^−^^7^	9.18 × 10^−^^4^	No
Endosulfan Sulphate	2.51 × 10^−^^9^	4.19 × 10^−^^5^	No	4.58 × 10^−^^7^	7.63 × 10^−^^4^	No

CDI: chronic daily intake, HQ: Hazard quotient.

## 2 Experimental design, materials and methods

Soil samples were taken from five locations situated in Machang, Kelantan of Peninsular Malaysia (Fig. 1). Agriculture practice in this area is paddy plantation. Each sampling point was geo-referenced using a handheld GPS Explorist 300 (Table 1). Samples were collected twice during September 2017 and February 2018 (*n* = 10). About 500 g soil samples (0–20 cm on top surface) were collected using a pre-cleaned plastic shovel, then placed on wrapped baked aluminum foil before being transferred into zip-locked polyethylene bags. Samples were placed into an ice-filled cool box before being transferred to the laboratory for further analysis.

The pH value of soil sample was measured using the slurry method. About 100 g of sample was diluted using deionised water with 1:1 ratio and stirred for 30 min before the pH value was taken. Organic carbon content and water content were obtained after calculating the difference of sample weight sample between before and after combustion at 375 °C and 105 °C, respectively [1]. About 200 g of soil sample was sieved using a calibrated mesh sieve to calculate the percentage of soil texture (clay, silt and sand) (Table 2). Classification of soil texture was constructed using a scale set by the International Soil Science Scheme as follows: sand (< 200 µm), silt (< 20 µm) and clay (< 2 µm), respectively [2].

The targeted compounds were hexachlorocyclohexane isomers (α, β, γ, δ), dichlorodiphenyl- trichloroethane family (4,4′-DDT, 4,4′-DDE, 4-4′-DDD), endosulfan (α, β,) and endosulfan sulphate. Soxhlet extraction (hexane: acetone 50:50 v/v) was used for isolation of the targeted compounds in 100 g sample, followed by a clean-up process using 200 mg 3 ml florisil column Agilent. Final determination was performed using gas chromatography-electron capture detector Varian CP-3800. Operating system for chromatographic separation followed the best condition obtained during our previous work [3]. Analysis was conducted in triplicate and the value was reported as the mean concentration. Actual concentration of pesticide residue (Table 3) was obtained through calculation using the linear regression method (7-concentration levels of mixture standard solution).

Non-dietary intake through ingestion route was calculated to evaluate the potential health risk of pesticide pollutants toward human. The formula used was adapted from literatures [4], [5]. Chronic daily intake was used as the main model, in which the estimation value was subject to adult (body weight 70 kg) and child (10 kg) exposure. The estimation risk of detected pesticide toward human was calculated using the hazard quotient formula. Reference dose for hexachlorocyclohexane isomers (3 × 10^−^^4^), dichlorodiphenyltrichloroethane family (5 × 10^−^^4^) and endosulfan sulphate (6 × 10^−^^4^) expressed in mg kg^−^^1^day^−^^1^ [4], [6]. The present status was in the range of 2.68 × 10^−^^6^–4.18 × 10^−6^ and 1.24 × 10^−^^4^–9.17 × 10^−^^4^ for adult and child exposures, respectively (Table 4). Good linearity was calculated at *R*^2^ = 0.990–0.997.
